# High-Fiber Diet during Pregnancy Characterized by More Fruit and Vegetable Consumption

**DOI:** 10.3390/nu13010035

**Published:** 2020-12-24

**Authors:** Rachelle A. Pretorius, Debra J. Palmer

**Affiliations:** 1 School of Medicine, University of Western Australia, 35 Stirling Highway, Crawley, WA 6009, Australia; rachelle.pretorius@research.uwa.edu.au; 2 Telethon Kids Institute, University of Western Australia, 15 Hospital Ave, Nedlands, WA 6009, Australia

**Keywords:** pregnancy, nutrition, dietary fiber, fruit, vegetables

## Abstract

Higher dietary fiber intakes during pregnancy may have the potential health benefits of increasing gut microbiome diversity, lowering the risk of glucose intolerance and pre-eclampsia, achieving appropriate gestational weight gain, and preventing constipation. In this observational cohort study, we have assessed the dietary fiber intakes of 804 women in late pregnancy, using a semi-quantitative food frequency questionnaire (SQ-FFQ). Overall, the median (interquartile range) dietary fiber intake was 24.1 (19.0–29.7) grams per day (g/day). Only 237/804 (29.5%) women met the recommended Adequate Intake (AI) of dietary fiber during pregnancy of 28 g/day. Women consuming the highest quartile of fiber intakes (34.8 (IQR 32.1–39.5) g/day) consumed more fruit, especially apples and bananas, than women consuming the lowest quartile of fiber intakes (15.9 (IQR 14.4–17.5) g/day). These women in the highest fiber-intake quartile were older (*p* < 0.01), more had completed further education after secondary school (*p* = 0.04)*,* and they also consumed more vegetables (67 g/day) compared to the women in the lowest fiber consumption quartile (17 g vegetables/day). Bread intakes of 39–42 g/day were consistent in quantities consumed across all four fiber-intake quartiles. Our findings suggest that antenatal education advice targeting increased fruit and vegetable consumption before and during pregnancy may be a simple strategy to achieve increased total dietary fiber intakes to reach recommended quantities.

## 1. Introduction

A woman’s dietary intake during pregnancy can have important implications for herself and her child’s future health [1]. However, despite adequate availability of a range of food choices in many developed countries, the increased availability of processed foods, urbanization, and lifestyle changes have contributed to high maternal intakes during pregnancy of energy-dense foods, inadequate intakes of micronutrient-rich and high-fiber foods, and predominance of western-style diet eating patterns [2,3]. The western-style diet is characterized by increased consumption of animal-derived protein (including processed meat), saturated fat, highly refined carbohydrate-based foods, and low-fiber intakes, and linked to numerous non-communicable diseases, including metabolic syndrome [2,4]

The benefits of higher dietary fiber intakes have been well established for many decades [5,6], and with recent heightened interest in the gut microbiome’s effects on various health outcomes, the spotlight has again been shone on dietary fiber intakes. Recently, Reynolds and colleagues (2019) summarized their findings from a systematic review and meta-analysis (*n* = 4635) where they identified that the benefit of dietary fiber intakes on total mortality, incidences of cardiovascular disease, type 2 diabetes and colorectal cancer is likely to be dose-response dependent [7]. Their analysis showed that for every 8 g increase of dietary fiber eaten each day, total deaths and incidence of coronary heart disease, type 2 diabetes and colorectal cancer decreased by 2–19% and with additional benefit likely to accrue with higher fiber intakes greater than 30 g per day [7]. 

During pregnancy, adequate dietary fiber intakes of 28 g/day are recommended in Australia [8]. In addition to increasing gut microbiome diversity, higher dietary fiber intakes during pregnancy may also reduce excessive weight gain, glucose intolerance, gestational hypertensive disorder and constipation [9,10,11,12]. Given the increased prevalence of these adverse health conditions during pregnancy in our community, we hypothesized that many women were consuming inadequate dietary fiber intakes during pregnancy. Hence, this observational study aimed to assess maternal dietary fiber intakes and determine the specific fiber-rich foods that were being consumed by pregnant women. 

## 2. Materials and Methods

### 2.1. Study Population

This observational cohort study was conducted in Perth, Western Australia, and approved by the Princess Margaret Hospital Human Research Ethics Committee (HREC approval numbers 768 EP and 1942 EP). The pregnant women were recruited from antenatal clinics and classes at local participating maternity hospitals in metropolitan Perth, Western Australia, between February 2003 and December 2013. The maternal participants’ inclusion criteria were maternal age ≥ 18 years, non-smoker in pregnancy, singleton pregnancy, gestation ≥ 36 weeks, and healthy pregnancy with no known complications (including no immunodeficiency, pre-eclampsia, or major congenital anomalies). All participants provided written informed consent. A total of 905 pregnant women in late pregnancy were recruited into this observational cohort. 

### 2.2. Maternal Demographics and Characteristics

Maternal demographic data was collected at 36–40 weeks gestation, including maternal age, education level, ethnicity, and parity. Maternal highest education level achieved was categorized into two groups: secondary school only; or further education completed after secondary school.

### 2.3. Maternal Dietary Assessment in Late Pregnancy

A maternal diet semi-quantitative food frequency questionnaire (SQ-FFQ) was administered at an appointment between 36–40 weeks gestation. The SQ-FFQ data was collected over a 10-year period and captured maternal dietary intakes from all months of the year and all seasons. The SQ-FFQ was developed and analyzed by The Cancer Epidemiology & Intelligence Division, Cancer Council Victoria, Australia [13,14]. The SQ-FFQ was used to assess the usual daily intake of foods and nutrients of 101 food items consumed over the previous month (32–36 weeks of gestation), providing an overall picture of food intake during late pregnancy. The SQ-FFQ was used to assess energy, carbohydrate, protein, fat, and dietary fiber intakes. The specific fiber-rich foods eaten were identified using the data on food items consumed, including fruit, vegetables, bread, cereal, and grain-based foods, legumes, and nuts. To further explore the typical fiber-rich foods consumed by the pregnant women, participants were divided into four quartiles (Q1–Q4), where Q1 contained participants who reported the lowest consumption of fiber and Q4 included the participants who ate the highest fiber intakes [15].

Under-reporting of energy intakes also affects the estimation of other nutrient intakes; therefore, the accurate assessment of energy intake is of particular importance in nutritional epidemiology [16]. Women who reported unrealistic energy intake estimates of below 4500 KJ or above 20,000 KJ per day were excluded to control for under or over-reporting of maternal dietary intakes, as per methodology in previous dietary analysis studies during pregnancy [17,18] 

### 2.4. Infant Birth Outcomes

After their estimated delivery date, the participating women were contacted by telephone, and infant birth outcomes of gestational age at birth, infant birth weight, and infant gender were collected. 

### 2.5. Statistical Analysis 

Statistical analyses were performed using the latest version of SPSS software (version 25.0 for Macintosh, SPSS Inc., Chicago, IL, USA). The normality of the data was tested by Kolmogorov–Smirnov Test. Descriptive statistics were used to summarize participant demographics. Characteristics were summarized as percentages and frequencies for categorical variables, mean (SD) for normally distributed continuous variables, and median (interquartile range (IQR)) for non-normally distributed continuous variables. Energy, carbohydrate, protein, fat and fiber intakes, maternal age, and infant gestational age at birth data were not normally distributed, whereas infant birth weight was normally distributed. Maternal demographic characteristics of participants were compared between quartiles of total dietary fiber intake, using Chi-square (categorical variables) and Kruskal-Wallis (continuous variables) where appropriate. Spearman’s rho (non-normal distribution) and Pearson (normal distribution) correlation were performed to investigate any associations between fiber intakes in late pregnancy and infant birth weight and gestational age at birth. Differences were regarded as significant at the level *p* < 0.05. 

## 3. Results

### 3.1. Study Population 

**Figure 1:**
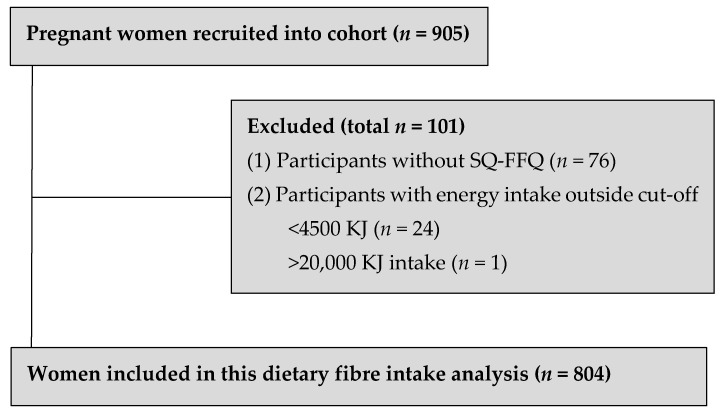
Study flow of participants.

Seventy-six women were excluded from this current dietary analysis due to missing SQ-FFQ data. Of the remaining 829 women, 25 women were excluded due to energy intake misreporting, resulting in a final 804 women as shown in Figure 1.

### 3.2. Participant Demographic Characteristics in Relation to Dietary Fiber Intakes

The demographical characteristics of the 804 women participants are shown in Table 1. The median maternal age was 32.9 (IQR 29.2–35.8) years, with a total maternal age range of 18–46 years. Among the women participants, 76.7% had completed further education after secondary school and half of the women were expecting their first child (49.7%).

Overall, the median (IQR) of dietary fiber intake was 24.1 (19.0–29.7) grams per day (g/day). Only 237/804 (29.5%) women achieved the recommended AI for pregnant women in Australia of 28 g/day of dietary fiber. Most of the women participants were Caucasian ethnicity (90.6%) and the remaining women came from many diverse ethnic communities, including Aboriginal/Torres Strait Islander, Maori/Pacific Islander, South-East Asian, Middle Eastern, and African. The average dietary fiber intake of the non-Caucasian women 24.8 g/day. 

Table 1 also summarizes the maternal characteristics according to the four dietary fiber-intake quartiles (Q1–Q4). The parity and ethnicity of women in the different fiber-intake quartiles were similar. Significant differences were found for maternal age (χ^2^ (3, *n* = 804) = 16.5, *p* < 0.01) and education level achieved (χ^2^ (1, *n* = 784) = 0.1, *p* = 0.04, phi = 0.1) between the different fiber-intake quartiles. Women in the highest fiber intakes quartile (Q4) were older with a median (IQR) of 32.9 (29.6–35.9) years, compared to the lowest fiber intakes quartile (Q1), median 31.0 (27.8–34.7) years. More women in Q4 had completed further education after secondary school (80.3%) compared to women in Q1 (70.1%).

The women’s energy, carbohydrate, protein, and fat intakes, were also calculated using the data from the SQ-FFQ. The women consumed a median energy intake of 8116.2 (IQR 6745.7–9945.9) KJ/day, carbohydrate intake of 225.4 (IQR 175.7–283.4) g/day, protein intake of 85.4 (IQR 69–102.1) g/day and fat intake of 78.6 (IQR 63.2–97.8) g/day.

### 3.3. Fiber-Rich Foods Consumed during Late Pregnancy

**Figure 2:**
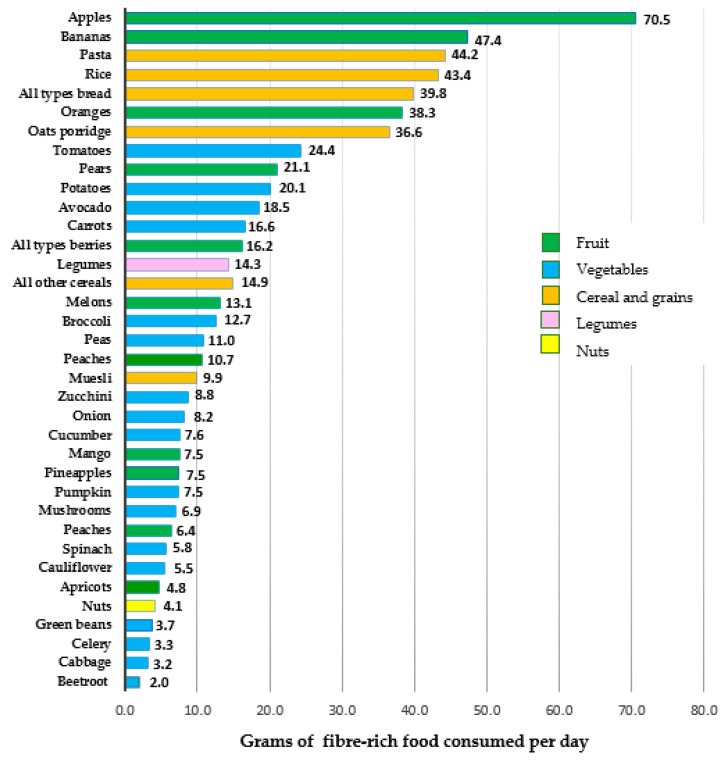
Fiber-rich foods (grams/day) consumed by women during late pregnancy.

The fiber-rich foods consumed by the pregnant women in this cohort in late pregnancy are summarized in Figure 2. A total of 37 fiber-rich foods were identified from the SQ-FFQ. These fiber-rich foods were categorized into fruit, vegetables, cereal and grain-based foods (consisting of all types of bread, cereals, rice and pasta), legumes, and nuts. Apples were the fiber-rich food most consumed by the pregnant women in this cohort, followed by bananas, pasta, rice and all types bread (including white fiber-rich bread, whole-grain bread, and rye bread). Although the women consumed various vegetables, no specific individual vegetables were featured in the top five fiber-rich food sources. Tomatoes and potatoes were among the vegetables most consumed (8th and 10th place respectively), followed by avocado, carrots, broccoli, and peas. The fruit least consumed by the women were apricots and peaches, whereas beetroot, cabbage, and celery were among the vegetables least consumed. Overall, the women consumed an average of 243.5 g/day of fruit, 188.8 g/day of cereal and grain-based foods, and 165.5 g/day of vegetables. The women consumed limited intakes of legumes (14.3 g/day) and nuts (4.1 g/day). 

In comparison to the Australian Dietary Guideline recommendations for fruit and vegetable (including legumes) consumption for pregnant women (2 serves of fruit and 5 serves of vegetables per day), the pregnant women in this study consumed on average 1.6 serves of fruit and 2.2 serves of vegetables per day. The recommended intake of fruit (2 serves per day) was achieved by only 24.8% (*n* = 200) of the women, and only 3.5% (*n* = 28) met the daily recommended vegetable intake (Table 2).

### 3.4. Top 10 Fiber-Rich Foods Consumed Based on Quartile Dietary Fiber-Intake Groupings

**Figure 3:**
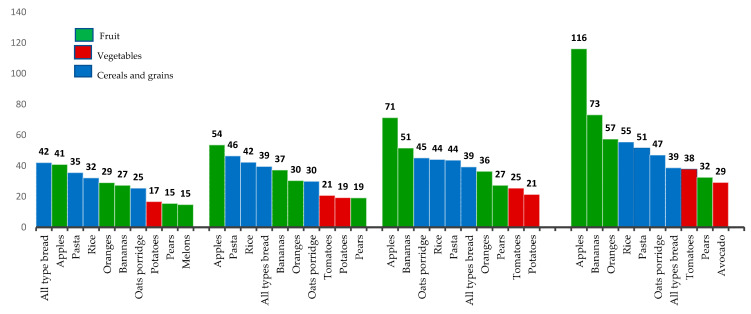
Ten most consumed fiber-rich foods by the women based on quartile fiber intakes.

Figure 3 summarizes the results from the 10 most consumed fiber-rich foods during late pregnancy based on the quartile (Q1–Q4) dietary fiber-intake groupings. Women in the highest fiber-intake (Q4) quartile consumed 116 g/day of apple, whereas women only consumed 41 g/day of apple in the lowest fiber quartile. Women in the high-fiber group also consumed more bananas (73 g/day) and oranges (57 g/day) than the women in the lowest quartile of fiber intake, who only consumed 27 g/day of bananas and 29 g/day of oranges. As expected the amount of fruit eaten increased from Q1 to Q4 dietary fiber intakes, with women consuming 278 g/day of fruit in Q4 compared to 127 g/day of fruit in Q1. Women in the high-fiber intake group (Q4) also consumed more vegetables (67 g/day), compared to the lowest fiber consumers (Q1) (17 g/day), who only ate potatoes in their top ten fiber-rich foods consumed. Bread intakes were consistent in quantity consumed across all fiber-intake quartiles (39–42 g/day)

### 3.5. Associations between Dietary Fiber Intake during Late Pregnancy and Infant Birth Outcomes

In this cohort comprised of 51.3% male infants, the mean (SD) infant birth weight was 3499.1 (±435) grams, and median gestational age at birth was 39.3 (IQR 38.6–40.1) weeks. We found no associations between maternal fiber quartile intakes in late pregnancy and infant gestational age or weight at birth, as reported in Table 3. 

## 4. Discussion

Higher maternal dietary fiber intakes during pregnancy can assist with appropriate gestational weight gain, reduce gestational glucose intolerance and hypertensive disorders, prevent constipation, and influence microbial gut diversity [10,12,19]. However, in our pregnancy cohort (*n* = 804), we found that only 29.5% of women were meeting the recommended AI for dietary fiber of 28 g/day. The median fiber and energy intakes in our cohort were 24.1 (IQR 19.0–29.7) g/day and 8116 (IQR 6745–9945) KJ/day, similar to 26.3 (IQR 20–33) g/day and 8280 (IQR 6718–10,004) KJ/day reported from another recent Australian cohort (*n* = 503 pregnant women) [20]. Hence as highlighted by these two cohorts, many Australian women appear to be consuming insufficient dietary fiber intakes during pregnancy. In comparison, higher dietary fiber intakes during pregnancy have been observed in women in Denmark (28 g/day) and Norway (32.2 g/day), and lower average intakes in the United States (19.8 g/day) and in the United Kingdom (17.2 g/day).

Epidemiological studies have associated higher consumption of fiber-rich fruit, vegetables, and whole-grain foods during pregnancy with reduced risks of adverse pregnancy and birth outcomes [21,22,23,24]. The Australian Dietary Guidelines recognize the importance of a high-quality diet during pregnancy, with dietary guidelines specifically targeted for pregnant women to improve maternal nutrition and provide the fetus with the best possible nutritional environment for growth and development in-utero [25]. The Australian Dietary Guidelines recommend 2 servings of fruit and 5 serves of vegetables (including legumes) each day during pregnancy [26]. Similar to previous studies, our dietary intake analysis demonstrated that pregnant women are not meeting nutritional guidelines [20,27,28,29,30]. In this study, the median fruit intake for the pregnant women was 1.6 serves per day, with only a quarter (24.8%) of the women meeting the target aim of two serves of fruit per day. Even more concerning was our finding that only 3.5% (*n* = 28) of the pregnant women in this study met the Australian Dietary Guidelines recommendation for 5 serves of vegetables (including legumes) per day [25]. Our findings for fruit intake were comparable to those from previous Australian-based studies in which the dietary intake of pregnant women was assessed [20,27,28,29]. In a recent observational study by Slater et al. (2020), using the Australian Eating Survey method to assess intakes of 503 pregnant women, the median fruit intake was 1.7 serves of fruit, with only 38.2% meeting the target of 2 serves per day [20]. Although the number of participants in our study meeting the recommended serving quantities of vegetables (3.5%) was lower than other Australian cross-sectional studies by Lee et al., Malek et al., and Slater et al. with 10%, 10.3% and 26.6%, respectively [20,27,28], it was similar to the percentage of women meeting recommended vegetable servings (2.0%) in a study by Mishra et al. published in 2016 [29].

Dividing women into quartiles based on their dietary fiber intakes provided insight into the dietary fiber-rich specific foods the women consumed. Women in the highest fiber-intake quartile group consumed more fruit and vegetables than women in the lowest fiber diet group. Interestingly, bread intake was consistent across all four fiber-intake quartiles (39–42 g/day, 1 serving of all types bread). Legume and nut intakes across all groups were limited, and hence pregnant women should be encouraged to consume more legumes and nuts as part of a balanced diet during pregnancy.

Our study results illustrate that apples and bananas are the fiber-rich foods most consumed by pregnant women. Apple and banana consumption has been associated with various health benefits, including improving cardiovascular health, maintaining ideal blood glucose levels, treating various gastrointestinal tract disorders, all-cause cancer mortality, weight management and diabetes [31,32,33,34,35]. In general, fiber from fruit and vegetables tend to be more fermentable, increasing gut microbial diversity and short-chain fatty acid production [15]. Dietary fiber sub-types, such as soluble fiber, insoluble fiber, resistant starch, and prebiotic fiber, each have a unique chemical structure, physical properties and health effects [36,37]. The level of ripeness and even cooking/cooling process may influence the nutritional content and chemical structure of resistant starch (classified into 5 classes, RS1–RS5) within the same fruit [38]. For example, the banana resistant starch content is known to decrease during the 4 ripening stages, from 38.28 g/100 g in stage 1 to 12.9 g/100 g in stage 4 [36,39,40]. In uncooked green banana flour the resistant starch content is 5.5–16.6 g/100 g, whereas green banana starch, cooked green banana flour and ripe bananas contain 34–67 g/100 g, 1.2 g/100 g and 7.8 g/100 g respectively [34]. Apples, the world’s second most consumed fruit after bananas, also contain various dietary fiber sub-types [32]. Apples contain about 2.2 g/100 g total fiber, of which 70% is insoluble, including cellulose and hemicellulose, and 30% is soluble, mainly pectin [31]. Pectin, the major component of soluble fiber in apples, affects gut transit time, gastric emptying, and nutrient absorption. Pectin also can modulate the gut microbiota and reduce serum triglyceride levels [41]. The different dietary fiber sub-types have varied structural functions which remain poorly understood, hence ongoing research is warranted to further understand the physiological roles and health benefits of specific dietary fiber sub-types.

Our study’s strengths include using the validated Australian SQ-FFQ [13,14], a well-accepted, practical, and affordable method for quantitative assessment of usual nutrient intake. The use of the SQ-FFQ enabled us to quantify total fiber intakes and describe the consumption patterns of specific fruit, vegetables, cereals and grain-based foods, nuts, and legumes during late pregnancy. To the best of our knowledge, we have reported for the first time the specific fiber-rich foods consumed by pregnant women and how these food types may differ between high- and low-fiber diets. Our study findings provide a novel insight into the specific fiber-rich foods that women consume during pregnancy, and highlight the limited consumption of vegetables that many women eat, especially those women in the lowest quartile dietary fiber intakes.

We do acknowledge that our findings are limited in the regard that we only assessed dietary intakes at one-time point in late pregnancy, and there may have been changes in fiber consumption between the first, second, and third trimesters. Further longitudinal studies are needed to identify any differences in fiber-rich food intakes throughout pregnancy. We also acknowledge that our study only captured dietary intakes from women in the Perth metropolitan area and did not include women from rural communities where consumption patterns may differ. Also, as most of our participants were of Caucasian ethnicity (90.6%), our results may have reduced generalizability to other cultural dietary consumption patterns.

Higher dietary fiber intakes can have health benefits to women during pregnancy, including lowering the risk of diabetes, pre-eclampsia, and constipation [10]. Thus, it remains a major concern that suboptimal dietary quality and low adherence to dietary guidelines have been consistently reported during pregnancy. Pregnancy is an important life stage and a ‘teachable moment’ for introducing positive changes in diet and lifestyle [42]. Our findings that the pregnant women in the high-fiber group (Q4) were older and had higher education level achievement were consistent with several other cohort studies, where increased fiber-rich fruit and vegetable consumption was associated with increased age and higher education level [6,43,44,45,46,47]. Our study supports the notion that educated women tend to make healthier food choices and are likely to consume diets approaching adequate recommended intakes [45]. In contrast, younger maternal age and socioeconomic disadvantage are associated with increased risk of lower nutritional intakes and subsequent increased risk of adverse maternal and infant health outcomes [48]. All women, irrespective of age and education level, should receive nutritional education to optimize their own and their infant’s health [49], but public health initiatives that focus on improving pregnant women’s healthy eating choices from more disadvantaged communities and younger women of childbearing age should be prioritized, if nutritional education resources are limited.

## 5. Conclusions

Adequate dietary fiber intakes during pregnancy are important for both maternal and infant health. Our results suggest that many pregnant women have insufficient dietary fiber intakes. Based on the dietary fiber-intake quartiles in our cohort, women eating high-fiber diets eat more fruit (mainly apples and bananas) and vegetables than women who eat a low-fiber diet. This increased knowledge of which specific fiber-rich foods pregnant women consume can help targeted healthy eating strategies and support women to optimize their fruit and vegetable intakes to reach the recommended intake of dietary fiber each day, especially during the critical period of pregnancy.

## Figures and Tables

**Table 1:** Participant demographic characteristics according to dietary fiber-intake quartiles (Q1–Q4).

Maternal Characteristics	All Participants (*n* = 804)	Q1 Fiber intake * 15.9 (14.4–17.5) *n* = 202	Q2 Fiber Intake * 21.4 (20.3–22.6) *n* = 202	Q3 Fiber Intake * 26.5 (25.2–27.6) *n* = 199	Q4 Fiber Intake * 34.8 (32.1–39.5) *n* = 201	*p* Value
Age (years) ^#^	32.9 (29.2–35.8)	31.0 (27.8–34.7)	33.0 (30.0–35.9)	33.3 (30.4–36.0)	32.9 (29.6–35.9)	<0.01
Parity (0) ^¥^	374/754 (49.6%)	102/187 (54.5%)	80/187 (42.8%)	96/188 (51.1%)	97/192 (50.5%)	0.14
Ethnicity (Caucasian) ^¥^	665/734 (90.6%)	161/177 (91.0%)	160/184 (87.0%)	172/184 (93.5%)	173/189 (91.5%)	0.18
Further education post-secondary school ^¥^	601/784 (76.7%)	138/197 (70.1%)	149/197 (75.6%)	15/192 (80.7%)	159/198 (80.3%)	0.04

**^¥^** Number (Percentage), ^#^ Median (IQR), * dietary fiber intake (g/day).

**Table 2:** Number (and %) of women meeting the Australian Dietary Guidelines daily recommended fruit and vegetable consumption.

Food Group	Recommended Daily Serving	Consumed Less Than Recommended	Meeting Recommendation and Consuming More Than Recommended
Fruit	2	604 (75.1%)	200 (24.8%)
Vegetables (and legumes)	5	776 (96.5%)	28 (3.5%)

**Table 3:** Infant birth outcomes for the four quartiles of maternal dietary fiber intake.

Infant Characteristics	All Participants *n* = 804	Q1 15.9 (14.4–17.5) * n = 202	Q2 21.4 (20.3–22.6) * n = 202	Q3 26.5 (25.2–27.6) * n = 199	Q4 34.8 (32.1–39.5) * n = 201	*p* Value
**Gender (male) ^¥^**	394/768 (51.3%) ^§^	101/192 (47.4%)	96/189 (51.0%)	100/194 (51.5%)	99/196 (50%)	0.56
Birth weight **	3499.1 (435.0) ^＊^	3466.8 (485.0)	3514.5 (418.3)	3489.5 (411.5)	3523.6 (422.0)	0.55
**Gestational age ^#^**	39.3 (38.6–40.1) ^★^	39.2 (38.4–40.3)	39.0 (38.6–40.0)	39.4 (38.6–40.1)	39.4 (37.7–40.3)	0.12

^¥^ Number (Percentage), ^#^ Median (IQR), ** Mean (SD), * Total dietary fiber (g/day). ^＊^ *N* = 772 available data on infant birth weight, ^§^ *n* = 768 available data on infant gender, ^★^ *N* = 761 available data on infant gestational age.
